# Combined Screening for Early Detection of Pre-Eclampsia

**DOI:** 10.3390/ijms160817952

**Published:** 2015-08-04

**Authors:** Hee Jin Park, Sung Shin Shim, Dong Hyun Cha

**Affiliations:** Department of Obstetrics and Gynecology, CHA Gangnam Medical Center, CHA University, Seoul 135-081, Korea; E-Mails: coolsome72@chamc.co.kr (H.J.P.); chadh001@chamc.co.kr (D.H.C.)

**Keywords:** pre-eclampsia, early prediction, maternal risk factors, mean maternal arterial pressure, ultrasound parameters, biomarker, pregnancy-associated plasma protein-A (PAPP-A), cell-free fetal DNA

## Abstract

Although the precise pathophysiology of pre-eclampsia remains unknown, this condition continues to be a major cause of maternal and fetal mortality. Early prediction of pre-eclampsia would allow for timely initiation of preventive therapy. A combination of biophysical and biochemical markers are superior to other tests for early prediction of the development of pre-eclampsia. Apart from the use of parameters in first-trimester aneuploidy screening, cell-free fetal DNA quantification is emerging as a promising marker for prediction of pre-eclampsia. This article reviews the current research of the most important strategies for prediction of pre-eclampsia, including the use of maternal risk factors, mean maternal arterial pressure, ultrasound parameters, and biomarkers.

## 1. Introduction

Pre-eclampsia (PE) is a multisystemic disorder that originates in early pregnancy and leads to considerable maternal morbidity and mortality [1,2,3]. Although there are still many unanswered questions, the pathophysiology of pre-eclampsia likely involves both maternal and fetal/placental factors. Abnormalities in the development of placental vasculature early in pregnancy is considered to be a primary cause of relative placental underperfusion/hypoxia/ischemia, which then leads to release of numerous factors into the maternal circulation that alter maternal systemic endothelial function and cause hypertension and other manifestations of the disease. Although abnormal spiral artery remodeling is widely regarded as a critical role, the underlying cellular and molecular mechanisms remain obscure [4].

PE can be classified into early and late onset, and it is widely accepted that these subtypes of PE represent different forms of the disease. Early-onset PE, requiring delivery before 34 weeks’ gestation, is commonly associated with intrauterine growth retardation (IUGR), abnormal uterine and umbilical artery Doppler waveforms, and adverse maternal and neonatal outcomes. In contrast, late-onset PE, with delivery at or after 34 weeks, is mostly associated with mild maternal disease and a low rate of fetal involvement. The perinatal outcomes of late-onset PE are usually favorable [5,6,7].

Early detection of PE would allow for planning of appropriate monitoring and for clinical management, following early identification of complications. Trials of prophylactic intervention for PE from mid-gestation have not been efficacious. However, the prediction of PE in early gestation may lead to more effective early prophylactic strategies [8].

For the last three decades, a number of research groups have investigated the value of low-dose aspirin as prophylactic use. Recent meta-analyses have suggested that, provided treatment is started at an early (<16 weeks’) gestation, there is a significant reduction in early-onset PE and that this is associated with a reduction in prevalence of perinatal death and morbidity [9,10,11,12]. Various national and international agencies currently recommend that women deemed to be at high risk of PE should be offered aspirin therapy [13,14]. This reinforces the need for early identification of high risk women with the objective of implementing targeted interventions for improving perinatal and maternal outcomes.

No single test has demonstrated a sufficient predictive value for PE to be of clinical use [15]. These tests appear to be most useful in combination with other parameters instead. Because of the heterogeneous nature of PE, a combination of two or more independent biomarkers, each reflecting a different pathophysiological process, should potentially increase the likelihood to derive suitable predictive algorithms [16]. Researchers still continue to search to identify a combination of tests that will work better than, or in association with, uterine artery Doppler to maintain a high sensitivity and improve specificity [17].

The most promising strategies for the prediction of PE involve multiparametric approaches, which use a variety of individual parameters in combination (e.g., as established in first-trimester aneuploidy screening). A combination of maternal risk factors, the uterine artery pulsatility index (PI), mean arterial pressure (MAP), and maternal serum pregnancy-associated plasma protein-A (PAPP-A), placental growth factor (PlGF), PP13, and fetal hemoglobin levels at 11–13 weeks’ gestation can be used to identify a high proportion of pregnancies at high risk for early-onset PE [18,19,20,21,22,23]. We reviewed the potential of novel biomarkers, such as cell-free fetal DNA generated by novel research strategies, to attempt to improve the predictive performance of the existing models.

## 2. Maternal Factors and History

Multiple factors, including previous PE, antiphospholipid syndrome, family history of PE, chronic kidney disease, insulin-dependent diabetes, multiple pregnancies, preexisting hypertension, and nulliparity are well-documented risk factors for PE [13]. Many guidelines in obstetrical practice currently recommend that low-dose aspirin therapy should be offered to women with high-risk factors of PE during pregnancy [13,14,24,25]. Although screening strategies for the early identification and subsequent prevention of PE have been suggested and maternal previous history of PE alone has a limitation in predicting PE, “Task force on hypertension in pregnancy” of the American college of obstetricians and gynecologists recently recommended the administration of daily low-dose (60–80 mg) aspirin beginning in the late first trimester only when women had a medical history of early-onset PE and preterm delivery at less than 34 weeks of gestation or PE in more than one prior pregnancy [25]. However, screening strategies using maternal factors and history alone for detection of PE only perform moderately well at best.

Screening with these factors as suggested by the National Institute for Health and Clinical Excellence results in a false-positive rate of 64.1%, with detection rates of 89.2%, 93.0%, and 85.0% for early-PE, late-PE, and gestational hypertension, respectively. In a prospective screening study of the National Institute for Health and Clinical Excellence, when these same factors were combined into an algorithm derived from multivariate analysis, they yielded better results with detection rates of 37%, 29%, and 21%, respectively, with a 5% false-positive rate. Although maternal factors and history alone have limitations in predicting PE, these can be potentially useful with different algorithms [26]. The limitations of using maternal factors alone to predict PE in primigravidae were shown by the multicenter, prospective Screening for Pregnancy Endpoints study, which determined that 9% of nulliparous women would be referred to specialist care, of whom 21% would develop PE [27].

Different subsets of factors are better at predicting early-onset PE (previous history of PE, black ethnicity, pre-existing hypertension, and previous use of ovulation inductors) than late-onset PE (maternal or family history of PE, black ethnicity, body mass index, and maternal age). With this approach to screening, the effects of variables are expressed as odds ratios for early-onset, late-onset, or total PE. This has led to the view that early- and late-onset PE may be different diseases. Pre-existing maternal subclinical endothelial dysfunction is likely to make a woman more vulnerable to poor placentation and subsequent placental dysfunction [28].

Development of PE is thought to include abnormal placentation and its vascular supply, Therefore, the evaluation of uterine artery blood flow resistance, besides maternal risk factors, is reasonable. Some recent reports have shown that, such assessment of risk is based on a combination of maternal history, blood pressure and uterine artery Doppler, the detection rate of early PE is higher than based on maternal history alone [26,29].

## 3. Uterine Artery Doppler

During normal pregnancy, invasive cytotrophoblasts of fetal origin remodel maternal spiral arteries, causing them to dilate into large flaccid vessels. This remodeling process leads to an increase in flow in the fetomaternal circulation, which is required for adequate placental perfusion. However, poor placentation with inadequate remodeling of the spiral arteries has been associated with development of PE, IUGR, and other associated complications [30,31,32].

Many studies have shown that increased impedance of blood flow in uterine arteries is associated with the development of PE and the results of previous first and second trimester Doppler studies are also compatible with histopathological findings of placentas from PE [33,34,35,36]. The state of a high resistance of the uteroplacental circulation can be measured noninvasively by uterine artery Doppler ultrasound [37]. Uterine artery Doppler screening is a useful screening program for prediction of PE. Color flow mapping is used to identify vessels, either transabdominally at the apparent crossover with the external iliac artery, or transvaginally lateral to the uterine cervix at the level of the internal cervical os [34]. Pulsed wave Doppler is then used. When three similar consecutive waveforms are obtained, the PI is measured and the mean PI of the left and right arteries is calculated. Peak systolic velocity should be greater than 60 cm/s to ensure that the arcuate artery is not being sampled instead of the uterine artery [36]. Most previous studies used uterine artery Doppler that was measured in the second trimester [38]. However, an increasing number of studies have shown the effectiveness of first-trimester uterine Doppler measurements for prediction of PE and IUGR [34]. A meta-analysis of 74 studies of PE (total of 79,547 singleton pregnancies) showed that uterine artery Doppler ultrasonography has a better performance in the second trimester than in the first trimester, and is useful for identifying severe or early-onset PE. Among low-risk women, an increased uterine artery PI in the second trimester has a sensitivity of 78% and specificity of 95% for detecting severe PE (positive likelihood ratio: 15.6; negative likelihood ratio: 0.23). In particular, an increased PI with notching in the second trimester is the best predictive marker in low- and high-risk patients [39].

More recently, 11 studies (43,122 women) evaluated the role of first-trimester uterine artery Doppler for the prediction of PE. They reported that abnormal uterine artery Doppler in the first trimester has a high specificity and low sensitivity in predicting early onset of PE. The overall sensitivity and specificity of uterine artery Doppler in the first trimester were 0.26 (95% confidence interval (CI) 0.24, 0.29) and 0.91 (95% CI 0.91, 0.91), respectively [40]. First-trimester uterine artery PI is affected by gestational age at screening, maternal weight, racial origin, and a history of preexisting diabetes mellitus. Therefore, this index should be expressed as a multiple of the median (MoM) after adjustment for these factors.

The MoM value of the uterine artery PI is significantly increased at 11–13 weeks’ gestation in women who subsequently develop early onset PE, and there is a significant negative linear correlation between the uterine artery PI MoM with gestational age at delivery [41]. The combination of the uterine artery PI in the first trimester and maternal factors may improve the detection rates from 36% to 59% and from 33% to 40%, with a false-positive rate of 5%, and from 51% to 75% and from 43% to 55%, with a false-positive rate of 10%, for PE requiring delivery before 34 and 37 weeks’ gestation, respectively. Finally, monitoring the uterine artery in the first trimester will enable clinicians to determine women at risk of developing early-onset PE and fetal growth restriction, and initiate preventive methods, such as aspirin therapy and regular fetal monitoring, to minimize adverse outcomes [42].

## 4. Placental Volume and 3D Power Doppler

Three-dimensional (3D) ultrasound can provide improved imaging of fetal anatomy compared with conventional 2D ultrasound. Specifically, novel assessment of the placenta by 3D ultrasound is more available than 2D ultrasound, including surface-rendering imaging and volume measurement. With the recent advances in 3D power Doppler ultrasound, more powerful evaluation of vascularization and blood flow of the placenta is available [43]. Several small studies have suggested that some parameters derived from 3D power Doppler of the placenta in the first trimester can predict adverse pregnancy outcomes, including PE and IUGR [44]. In a recent prospective nonintervention study of 308 women at 11 to 13 + 6 weeks’ gestation, 17 women who developed PE had a significantly reduced index of vascularization in 3D Doppler. However, the blood flow index was not different in women who developed PE. Placental volume is not appropriate for early prediction of PE or IUGR [45]. In the future, more large-scale studies are required to determine the potential of 3D ultrasound in prediction of PE.

## 5. Blood Pressure and Mean Arterial Pressure (MAP)

Small changes in blood pressure are a marker of risk of developing PE [28]. Women who subsequently develop PE have higher systolic blood pressure and MAP before the onset of clinical disease [31]. MAP is calculated by dividing the sum of the systolic and twice the diastolic blood pressure by three, and is thus easily measurable. A meta-analysis from 2008, including more than 60,000 women with 3300 cases of PE, showed that MAP was more predictive of PE among low-risk women in the first or second trimester than either systolic or diastolic readings alone [46].

For high-risk women, diastolic blood pressure measured between 13 and 20 weeks of gestation is the most predictive parameter for PE (positive likelihood ratio of 2.8). A prospective study in 5590 women with singleton pregnancies showed that a combination of maternal risk factors and MAP measured between 11 and 13 + 6 weeks of gestation was more predictive of PE (area under the curve (AUC): 0.852) than either alone (AUCs: 0.801 and 0.734, respectively) [47]. Another study on maternal characteristics and MAP-1 (11–13 weeks), with a false-positive rate of 10%, showed detection rates of 74.3%, 62.9%, and 49.3% in early PE, preterm PE, and total PE, respectively [38]. In screening of MAP-1 and MAP-2 (20–24 weeks’ gestation) at a false-positive rate of 10%, the detection rates were 84.3%, 65.7%, and 52.5%, respectively. Performance of screening for PE by MAP was the best when measurements were taken at 11–13 and 20–24 weeks’ gestation than at only one of these gestational ranges [48].

Based on the above mentioned studies, a two-stage strategy for identification of pregnancies at risk of PE has been proposed [49]. The goal of the first stage, at 11–13 weeks, is to predict preterm PE, because the prevalence of this condition can be substantially reduced by the prophylactic use of low-dose aspirin started before 16 weeks’ gestation [9,12,41,47,50]. The second stage, at 30–33 weeks, is focused on the effective prediction of PE requiring delivery at or after 34 weeks’ gestation. This is because close monitoring for earlier diagnosis of the clinical signs of PE could potentially improve perinatal outcome through interventions, such as administration of antihypertensive medication and early delivery [51].

A study on 35,215 pregnancies at 11–13 weeks of gestation used a survival time model to predict PE. This study reported that with combined screening by maternal factors (the uterine artery PI and MAP) at a false-positive rate of 5%, approximately 80% of PE patients deliver before 34 weeks’ gestation and 40% of PE patients deliver at 34–37 weeks’ gestation [50]. In a recent prospective study on PE at 30–33 weeks of gestation, combined screening by the maternal factors of the uterine artery PI and MAP identified 90% of pregnancies developing PE and requiring delivery within the subsequent 4 weeks. The authors concluded that the performance of combined screening at 30–33 weeks’ gestation for PE delivering at 34–37 weeks appears to be superior to that achieved by screening at 11–13 weeks’ gestation [52].

There is a strong association between the uterine artery PI and MAP in PE and unaffected pregnancies. When combining the uterine artery PI and MAP in calculating the specific risk for PE, correlation factors should be considered to avoid over or underestimating the contributions from each marker for providing accurate risk assessment for PE. Estimated detection rates of PE requiring delivery before 34, 37, and 42 weeks’ gestation in screening by maternal factors are 80%, 55%, and 35%, respectively, at a false-positive rate of 5%, and 90%, 72%, and 57%, respectively, at a false-positive rate of 10% [42].

## 6. Biomarkers

### 6.1. Angiogenic Factors

#### 6.1.1. Pro-Angiogenic Markers

##### Vascular Endothelial Growth Factor and Placental Growth Factor (PlGF)

Angiogenesis requires the complex interplay between the pro-angiogenic factors vascular endothelial growth factor (VEGF) and PlGF, with their cognate receptors VEGF receptor-1 (VEGFR-1, alternatively called fms-like tyrosine kinase-1 (Flt-1)) and VEGFR-2 [53].

Median levels of serum PIGF show a curvilinear relationship with gestational age with an increase in the first and second trimesters. PIGF levels reach a maximum at approximately 30 weeks’ gestation and subsequently decrease [54]. PlGF is a member of the vascular endothelial growth factor family and is implicated in angiogenesis and trophoblastic invasion of the maternal spiral arteries. Maternal serum levels of PlGF at 11–13 weeks’ gestation are decreased in pregnancies with fetal aneuploidies and those with impaired placentation, resulting in PE and delivery of small-for-gestational-age neonates. Serum PIGF levels are also reduced in the second and third trimesters in pregnancies with development of PE or in women who deliver small-for-gestational-age neonates [54].

#### 6.1.2. Anti-Angiogenic Markers

##### Serum Soluble Flt-1

Soluble Flt-1 (sFlt-1) is a truncated splice variant of the membrane-bound Flt-1. This splice variant circulates freely in the serum, where it binds and neutralizes VEGF and PlGF. Several studies have demonstrated an association between increased sFlt-1 levels and PE [55]. Levels of sFlt-1 levels begin to rise as early as five weeks before the onset of PE and they remain elevated compared with those in unaffected women [56]. In the decade since Maynard *et al.* [57] reported that excessive placental production of sFlt-1 (an antagonist of VEGF and PlGF) contributes to the pathogenesis of PE, extensive research has been published showing the usefulness of angiogenic markers in diagnosis and subsequent prediction and management of PE and placenta-related disorders. PlGF circulates free or in complexes with sFlt-1. The mechanistic role of PlGF in the pathogenesis of PE was first described by Zhou *et al.* [58] in 2002. The clinical utility of PlGF in screening for PE appears to be confined to early-onset disease [59,60,61]. Defective early placentation with impaired trophoblast invasion and restricted remodeling of the spiral arteries are central to the pathogenesis of early-onset PE, resulting in reduced uteroplacental perfusion. The combination of second- and third-trimester sFlt-1/PlGF ratios yields a detection rate of 87.5% at a fixed false-positive rate of 10% for early prediction of PE in a low-risk population [62].

##### Soluble Endoglin

Soluble endoglin (sEng) is a truncated form of receptor for transforming growth factor (TGF)-β1 and TGF-β2. This receptor is a potential anti-angiogenic factor, which interferes with binding of TGF-β1 to its receptor, and thereby affects production of nitric oxide, vasodilation, and capillary formation by endothelial cells *in vitro* [63]. In animal models, sEng, another antiangiogenic protein, acts together with sFlt-1 to induce a severe PE-like disease [64].

### 6.2. PAPP-A

PAPP-A is a large highly glycosylated protein that is produced by developing trophoblast cells. PAPP-A has been shown to be a syncytiotrophoblast-derived, insulin-like growth factor binding protein protease [65]. The insulin-like growth factor system is believed to play an important role in placental growth and development. Therefore, unsurprisingly, low serum PAPP-A levels are associated with a higher incidence of PE. Increased maternal serum PAPP-A levels have been observed in established PE [66,67,68]. A multicenter study of 8839 women demonstrated a significant relationship between PAPP-A levels at or below the 5th percentile and IUGR preterm delivery, PE, and stillbirth [69].

Predictive models for estimating individualized risk estimates for late-onset PE are based on a combination of first-trimester levels of PAPP-A and the second-trimester sFlt-1/PlGF ratio (detection rate of 87.5% at a fixed false-positive ratio of 5%) [62]. In pregnancies with development of PE, the maternal serum concentrations of PlGF and PAPP-A are reduced. These proteins are produced by the trophoblast, and their reduced maternal serum concentrations presumably reflect impaired placentation [37].

### 6.3. Inhibin-A and Activin-A

Inhibin-A and activin-A are glycoproteins and members of the TGF-β family. Both of these glycoproteins are largely released by the fetoplacental unit during pregnancy. Inhibin-A has an important endocrine role in the negative feedback of gonadotropins and activin-A is involved in various biological activities [70]. The concentrations of inhibin-A and activin-A, which are also produced by trophoblasts, are increased in women who will have PE, which may reflect a placental compensatory mechanism to promote trophoblastic invasion in cases where this process is impaired [69]. Inhibin-A as a predictor of PE has shown inconsistent results. Although an increased serum inhibin-A level is significantly associated with subsequent PE, inhibin-A levels have poor sensitivity for predicting PE [70,71]. Other studies have reported that mid-trimester inhibin-A levels are the best predictor of PE among multiple markers for Down syndrome screening [72,73].

Differences in activin-A levels are greater in late-onset PE than in early-onset PE, while the opposite is the case in other markers (PIGF, placental protein-13 (PP-13), inhibin-A, sEng, pentraxin-3, and P-selectin) [50]. Inhibin-A and activin-A have been shown to be increased prior to 14 weeks in PE pregnancies [74,75].

### 6.4. PP-13

PP-13 is a member of the galectin super-family (defined as galectin 13), a family of carbohydrate-binding proteins called b-galactoside-specific lectins in the syncytiotrophoblast [76,77]. Serum PP13 gradually increases to double-to-triple values before delivery in normal precnancies [78]. From as early as 5–7 weeks of gestation, serum PP13 levels in PE are significantly lower than in normal pregnancies [79,80]. Increased shedding of subcelluar necrotic microparticles (STBM) is most likely a source of high concentration of PP13 into maternal blood as PE progresses. The severity of the signs of PE is proportional to the increase of PP13 from first to third trimester [79,80]. For prediction of PE, combining PP13 with additional markers increased the DR [18,19,20]. When PP-13 was expressed as multiples of gestational age-specific medians in the control subjects, the MoM value was 0.2 for PE and the corresponding sensitivity was 79% with a specificity of 90% [81]. Recently, Gonen *et al.* have reported that combining PP13 with patient demography and history and with MAP improved prediction reaching 92% DR at 12% FPR [82].

### 6.5. A Disintegrin and Metalloprotease 12

A disintegrin and metalloprotease 12 (ADAM12) is a protease for insulin-like growth factor binding proteins. Low levels of ADAM12 reflect an increased amount of insulin-like growth factor in the bound state, and this is then unavailable to promote placental growth and development [83,84]. Recent studies have evaluated the association between low levels of ADAM12 and PE, and have produced conflicting results [85,86,87]. Spencer *et al.* demonstrated only a modest predictive efficiency of ADAM12 for PE with an AUC of 0.694 for ADAM12 alone and an AUC of 0.714 when ADAM12 and PAPP-A were combined [85]. Katherine *et al.* reported that median ADAM12 levels were significantly lower in patients who developed PE compared with those who did not (0.81 *vs.* 1.01 MoMs; *p* < 0.04), but ADAM12 was not sufficiently predictive of PE, resulting in an AUC of 0.58 (95% CI: 0.50, 0.67) [88].

### 6.6. Cystatin C

Cystatin C is an established marker for renal function, increasing as the glomerular filtration rate falls [89]. Placental expression of cystatin C is increased at the mRNA and protein levels in PE, suggesting increased synthesis and secretion of cystatin C protein. This could contribute to elevated maternal plasma cystatin C levels that are observed in PE [90]. Median cystatin C concentrations in the first trimester of pregnancy are significantly higher in women who subsequently develop PE (median, 0.65 mg/L) compared with those with a normal pregnancy (median, 0.57 mg/L, *p* = 0.0001) [91].

### 6.7. Pentraxin 3

Pentraxin 3 (tumor necrosis factor-stimulated gene-14) belongs to the same family as C-reactive protein and serum amyloid P component. Pentraxin 3 consists of 381 amino acids [92]. The maternal inflammatory response in established PE results in increased levels of pentraxin 3, an inflammatory marker from the same molecular class as C-reactive protein [93].

### 6.8. P-Selectin

P-selectin is a member of the selectin family of cell surface adhesion molecules. P-selectin is expressed by platelets and endothelial cells upon activation. This cell surface adhesion molecule plays a crucial role in inflammatory reactions by supporting recruitment and activation of circulating leucocytes, and in coagulation through generation of leukocyte-derived “bloodborne” tissue factor [94,95]. P-selectin is rapidly shed from the cellular membrane of activated platelets, and this release is suggested to contribute to most of the soluble isoform of the molecule that is found in plasma [96]. PE is associated with extensive platelet activation [97,98,99]. P-selectin-exposing micro-particles with procoagulant activity, released from activated platelets, have been detected in the peripheral blood of women with PE [100,101]. In addition, soluble P-selectin has been repeatedly, though not constantly, observed in higher amounts in serum or plasma of patients with PE [102,103,104,105]. However, a recent study reported that P-selectin, catalase, and superoxide dismutase were not significantly different between PE (case group) and normotensive pregnant women (control group) (*p* > 0.05) [106]. Because previous studies have shown inconsistent results, more in-depth research should be conducted before adopting P-selectin as a predictive marker for PE.

### 6.9. Fetal Hemoglobin

Extracellular HbF (fetal hemoglobin) has recently been suggested as a new predictive biomarker for pre-eclampsia and has also been hypothesized to be a causative factor [107,108]. Centlow *et al.* found an upregulation of HbF genes and accumulation of extracellular HbF in the vascular lumen in PE placentas [109]. Furthermore, the heme scavenger and antioxidant alpha(1)-microglobulin (A1M) increases in parallel with fetal hemoglobin [107]. After an unexplained placental hypoxia, an upregulation of placental HbF genes and proteins induce the formation of ROS, oxidative damage and leakage of the feto-maternal barrier in PE [107]. A defect placental hematopoiesis induces leakage of the feto-maternal barrier that results in endothelial dysfunction, hypertension, and proteinuria, which are all hallmarks of pre-eclampsia [107]. Looking at recent studies in which fetal hemoglobin was identified as a predictive first- and second-trimester biochemical markers for PE, Anderson *et al.* found significantly elevated levels of HbF and A1M in the PE group [21,22,23].

### 6.10. Genetic Markers of Pre-Eclampsia

The mode of inheritance of predisposition to pre-eclampsia is likely to be polygenic and influence from multifactorial pathogens. Candidate genes from various biological pathways involving the immune system, control of vascular resistance, blood coagulation, as well as those involved in cell signaling pathways and metabolic processes, have been the subject of many genetic association studies because of their putative roles in causing pre-eclampsia and its complications [110]. Some systematic reviews and meta-analysis have evaluated the association between maternal candidate genes and PE.

In Human Genome Epidermiology (HuGE) Review, Meta-analysis showed a higher risk of severe pre-eclampsia with coagulation factor V gene (proaccelerin, labile factor) (F5) polymorphism rs6025 (odds ratio = 1.90, 95% confidence interval: 1.42, 2.54; 23 studies, I2 = 29%), coagulation factor II (thrombin) gene (F2) mutation G20210A (rs1799963) (odds ratio = 2.01, 95% confidence interval: 1.14, 3.55, 9 studies, I2 = 0%), leptin receptor gene (LEPR) polymorphism rs1137100 (odds ratio = 1.75, 95% confidence interval: 1.15, 2.65; 2 studies, I2 = 0%), and the thrombophilic gene group (odds ratio = 1.87, 95% confidence interval: 1.43, 2.45, I2 = 27%) [110].

Seven genetic variants were found to be associated with pre-eclampsia in 2012. Genetic variants in or near the ACE (angiotensin-converting enzyme), CTLA4 (cytotoxic T-lymphocyte-associated protein 4), F2 (factor 2), FV (factor V, two variants), LPL (lipoprotein lipase), and SERPINE (serine peptidase inhibitor) 1 genes were associated with pre-eclampsia. The results of this meta-analysis suggest that the following systems may play a role in the pathogenesis of pre-eclampsia: the renin–angiotensin system, coagulation and fibrinolysis, lipid metabolism and inflammation [111]. Future genome wide association studies (GWAS) are warranted to investigate single nucleotide polymorphisms (SNPs) identified as the genetic risk factor for PE.

### 6.11. Cell-Free DNA

The immunostimulatory properties of cell free DNA have been known for over 50 years [112]. Human fetal DNA triggers *in vitro* activation of NF-κB, with resultant proinflammatory IL-6 production in both a human B-cell line and peripheral blood mononuclear cells from both pregnant and nonpregnant donors [113]. Administration of human fetal (but not adult) DNA into pregnant BALB/c mice causes fetal resorption with increased levels of tumor necrosis factor-α and IL-6 and infiltration by inflammatory cells in the placental bed [113]. Fetal DNA originates from the placenta, and placental-specific messenger RNA molecules are also easily detected in maternal plasma. There is also a graded response between the quantity of fetal DNA and the risk of developing PE [114], and hence, highest levels in women with HELLP syndrome [115,116]. The increase in shed DNA in PE reflects the increased hypoxic cell death. Levels of DNA released into supernatants from placental explants are increased when exposed to reduced oxygenation [116]. Thus, placental DNA released into the maternal circulation could be linking factor in the systemic inflammatory response of PE. The value of cell-free fetal DNA (cffDNA) in maternal plasma as an indicator of PE was first reported by Lo *et al.* in a small-scale study [117]. They found that in 20 PE women and 20 gestational age-matched controls in the third trimester, plasma cffDNA was increased approximately five-fold in women with PE. Currently, several multicenter studies are being performed to confirm the predictive value of cffDNA for predicting and monitoring PE in combination with other potential markers (e.g., P-selectin, PAPP-A, PP-13, sFlt-1, sEng, and PlGF) [118]. A recent systematic review reported that of 13 studies, 11 showed an increase in cffDNA in pregnant women who subsequently developed PE. In addition, all four studies that analyzed early-onset or severe PE showed significantly elevated cffDNA levels prior to disease onset [119].

RASSF1A is a tumor suppressor gene that is frequently inactivated by promoter hypermethylation in various tumors [120]. Because the placenta appears to be the only non-malignant tissue showing a densely methylated profile of RASSF1A, quantitation of hypermethylated RASSF1A can be used as an epigenetic marker for cffDNA [121]. Papantoniou *et al.* reported that cell-free DNA (cfDNA) and cffDNA levels from blood samples obtained at 11–13 weeks of gestation. They were significantly increased in women who developed PE compared with those with uncomplicated pregnancies (median cfDNA: 9402 *vs.* 2698 gΕq/mL; median cffDNA: 934.5 *vs.* 62 gΕq/mL, respectively). Following operating characteristic curve analysis, cutoff values of 7486 gΕq/mL for cfDNA and 512 gΕq/mL for cffDNA were chosen. These provided a sensitivity of 75% and 100% and a specificity of 98% and 100%, respectively, to identify women at risk for PE [122].

Cell-free fetal DNA quantification is a promising marker for prediction of PE. However, because of the heterogeneity in published studies, a precise conclusion about the statistical and clinical relevance of this potential marker cannot be made.

MicroRNA (miRNA) are noncoding RNA transcripts, that provide critical posttranscriptional regulation of gene expression in both health and disease through sequence-specific binding to the 3ʹ-untranslated region of target messenger RNA (mRNA) transcripts [123]. Recent studies have identified an abundance of miRNA in the healthy term placenta [124,125,126] and alterations of the miRNAome in cases of placental insufficiency highlighting a role for miRNA signaling in the development of PE [124,125,126,127,128,129]. Using qRT-PCR, Murphy *et al.* have identified increases in expression of seven maternal plasma miRNA (miR-98, miR-222, miR-210, miR-155, miR-296, miR-181a, and miR-29b) in patients with severe PE [130]. They have also demonstrated that differential expression of these miRNA at the time of pregnancy and PE are largely resolved one year postpartum in the maternal circulation [130]. Recent researches of placenta-specific miRNAs in the maternal circulation [131,132,133,134] have emphasized their value as predictive markers for placental insufficiency.

## 7. First Trimester Combined Screening

Maternal serum levels of PAPP-A and PlGF are two biochemical markers that have been extensively investigated and have shown promising results in early prediction of PE [37,50,54,62,66,67,68,69,135]. In screening by fetal nuchal translucency thickness (NT), fetal heart rate, free β human chorionic gonadotropin (β-hCG) and PAPP-A, using specific algorithms for trisomy 21 and trisomies 18 and 13 at the risk cutoff of 1:100, the estimated detection rate is 87.0% for trisomy 21 and 91.8% for trisomies 18 and 13, at a false-positive rate of 2.2% [136]. Addition of PLGF, α fetoprotein, and the ductus venosus PI for veins increases the detection rate to 93.3% for trisomy 21 and 95.4% for trisomies 18 and 13, and reduces the false-positive rate to 1.3%. PAPP-A and PIGF are useful in screening for aneuploidies in combination with maternal age, fetal NT, and maternal serum β-hCG at 11–13 weeks’ gestation [136]. If the serum markers that were investigated in this study [136] prove to have predictive value for PE apart from Down syndrome screening, PE and Down syndrome could be screened by a combination strategy at the same time in the first trimester.

Three studies derived from prospective first-trimester screening for adverse obstetric outcomes in the UK by the Fetal Medicine Foundation have reported the superiority of multiple biomarkers in the prediction of PE. In the first study, the combination of maternal characteristics and history, including the uterine artery PI, MAP, and serum PAPP-A, PIGF, PP-13, inhibin-A, activin-A, sEng, pentraxin-3, and P-selectin levels, were obtained from case-control studies. Algorithms that combine maternal characteristics and biophysical and biochemical tests at 11–13 weeks’ gestation could potentially identify approximately 90%, 80%, and 60% of pregnancies that subsequently develop early (<34 weeks), intermediate (34–36 weeks), and late (≥37 weeks) PE, respectively, at the false-positive rate of 5% [50].

The other two studies used competing risk models by a novel Bayes-based method that combines prior information from maternal characteristics and medical history, such as the uterine artery PI, MAP, and maternal serum PAPP-A and PlGF at 11–13 weeks’ gestation. This method can identify a high proportion of pregnancies at high risk for early-onset PE [41,61]. In the second study, screening by maternal characteristics with the uterine artery PI and MAP detected 90% of PE cases requiring delivery before 34 weeks’ gestation and 57% of all PE cases at a fixed false-positive rate of 10% [41]. In the third study, in screening for PE requiring delivery before 34 weeks’ gestation, the detection rate at a false-positive rate of 10% was approximately 50% by maternal characteristics, and improved to approximately 90% by the addition of biophysical markers and to approximately 75% by the addition of biochemical markers [61]. This detection rate improved to more than 95% by an algorithm combining maternal factors, biophysical markers, and biochemical markers (PAPP-A and PlGF) (Table 1).

**Table 1:** Algorithms for the prediction of early and late preeclampsia.

Study	Parameters	Early PE		Late PE	
		DR (%) for an FPR of			
		5%	10%	5%	10%
Akolekar *et al.* [50] 2011	A priori, MAP, UtA, PAPP-A, PlGF, PP13, sEng, inhibin A, activin A, PTX3, and P-selectin	91	95	61 ^a^–79 ^b^	71 ^a^–88 ^b^
Wright *et al.* [41] 2012	A.priori, UtA, MAP	80	90	55	72
Akolekar *et al.* [61] 2013	A priori, MAP, UtA, PAPP-A, and PlGF	93	96	38 ^a^–61 ^b^	54 ^a^–77 ^b^
Crovetto F *et al.* [137] 2015	A priori, MAP, UtA, PlGF, and sFlt-1	88	91	68	76

PE, preeclampsia; DR, detection rate; FPR, false-positive rate; n.a., data not available; A priori, maternal a priori risk; MAP, mean arterial pressure; UtA, uterine artery Doppler; PAPP-A, pregnancy-associated plasma protein; PlGF, placental growth factor; PP13, placental protein 13; sEng, soluble endoglin; PTX3, Pentatrexin 3; sFlt-1, soluble Fms-like tyrosine kinase-1; ^a^ This category was subdivided in late PE with delivery >37 weeks; ^b^ This category was subdivided in intermediate PE with delivery between 34 and 37 weeks.

The Screening for Pregnancy Endpoints study presented a series of models combining biomarkers and clinical and ultrasound data that predict the risk of PE and its different subphenotypes in an international pregnancy cohort of low-risk nulliparous women (*n* = 5623) [137]. The authors performed univariate analysis of 47 biomarkers and predictive models were constructed for PE, term PE (gestation at delivery ≥37 weeks), preterm PE (gestation at delivery <37 weeks), and early-onset PE (gestation at delivery <34 weeks). The final model for the prediction of PE included PIGF, MAP, and body mass index at 14 to 16 weeks’ gestation, as well as consumption of three or more pieces of fruit per day and the mean uterine artery resistance index through multivariable modeling. Neither model included PAPP-A, previously reported to predict PE significantly in populations of mixed parity and risk. In nulliparous women, combining multiple biomarkers and clinical data provided modest prediction of PE [137].

Logistic regression predictive models were developed for early- and late-onset PE in a Spanish prospective cohort of 9462 pregnancies undergoing first-trimester screening. The best model for early PE (*n* = 57, 0.6%) included a priori risk, MAP, the uterine artery PI, PlGF, and sFlt-1. For early PE, this model achieved detection rates of 87.7% and 91.2% and false-positive rates of 5% and 10%, respectively. For late-onset PE (*n* = 246, 2.6%), the best model included the a priori risk, MAP, the uterine artery PI, PlGF, and sFlt-1. This model achieved detection rates of 68.3% and 76.4% at false-positive rates of 5% and 10% [135]. In this study, angiogenic factors increased the detection rate by 11% for early-onset PE and by 20% for late-onset PE at a false-positive rate of 10%. The results for early-onset PE in this study [136] are similar to previous studies [50,61], but the improvement observed in the prediction of late-onset PE is better than that in previous studies [50,61].

## 8. Conclusions

Current understanding of the pathophysiology of PE suggests that it may be a collection of syndromes with different precipitating factors and outcomes [138]. The heterogeneous syndrome of PE, with its complex pathogenesis [139], is restrictive for using a single clinical risk factor or biomarker in early pregnancy for discriminating women destined to develop PE. Prediction of PE is widely accepted to rely on multi-parametric approaches because no single screening test provides a reasonable risk assessment [140]. There are several helpful markers for PE and the reliability of these markers provides clinicians with confidence in predicting PE at the first trimester. The discovery of cffDNA testing in maternal plasma will revolutionize current approaches to aneuploidy screening [141]. Levels of cffDNA are clearly increased prior to the onset of the clinical symptoms of pregnancy-related complications, such as PE, IUGR, preterm labor, placental previa, and hyperemesis gravidarum [142]. Recent studies [130,131,132,133,134] have highlighted placental-specific miRNAs as potent predictive markers in syndromes of placental insufficiency. Further and more intensive research is required to elucidate genetic markers for predicting PE in early gestation. Large-scale, multicenter, multi-ethnic, prospective trials with women considering different risks of developing PE are required to propose an ideal combination of markers for routine screening.
